# Protein engineering of *Saccharomyces cerevisiae* transporter Pdr5p identifies key residues that impact *Fusarium* mycotoxin export and resistance to inhibition

**DOI:** 10.1002/mbo3.381

**Published:** 2016-06-04

**Authors:** Amanda B. Gunter, Anne Hermans, Whynn Bosnich, Douglas A. Johnson, Linda J. Harris, Steve Gleddie

**Affiliations:** ^1^ Ottawa Research and Development CentreAgriculture and Agri‐Food CanadaOttawaONK1A 0C6Canada; ^2^ Ottawa‐Carleton Institute of BiologyUniversity of OttawaOttawaONK1N 6N5Canada

**Keywords:** ABC transporters, deoxynivalenol, drug resistance, enniatin, *Fusarium graminearum*, yeast

## Abstract

Cereal infection by the broad host range fungal pathogen *Fusarium graminearum* is a significant global agricultural and food safety issue due to the deposition of mycotoxins within infected grains. Methods to study the intracellular effects of mycotoxins often use the baker's yeast model system (*Saccharomyces cerevisiae*); however, this organism has an efficient drug export network known as the pleiotropic drug resistance (PDR) network, which consists of a family of multidrug exporters. This study describes the first study that has evaluated the potential involvement of all known or putative ATP‐binding cassette (ABC) transporters from the PDR network in exporting the *F. graminearum* trichothecene mycotoxins deoxynivalenol (DON) and 15‐acetyl‐deoxynivalenol (15A‐DON) from living yeast cells. We found that Pdr5p appears to be the only transporter from the PDR network capable of exporting these mycotoxins. We engineered mutants of Pdr5p at two sites previously identified as important in determining substrate specificity and inhibitor susceptibility. These results indicate that it is possible to alter inhibitor insensitivity while maintaining the ability of Pdr5p to export the mycotoxins DON and 15A‐DON, which may enable the development of resistance strategies to generate more *Fusarium*‐tolerant crop plants.

## Introduction

*Fusarium graminearum* is an economically important fungal pathogen of cereal crops. In North America, it is the predominant causal agent of gibberella ear and stalk rot in maize and fusarium head blight (FHB) in wheat and barley (McMullen et al. 2012; Mesterházy et al. 2012). The infection of crops by *F. graminearum* reduces yield and quality and often leads to grain contamination by the trichothecene mycotoxin deoxynivalenol (DON) and the acetylated derivatives 3‐acetyl‐DON (3A‐DON) and 15‐acetyl‐DON (15A‐DON). Trichothecenes interfere with numerous cellular processes in eukaryotic cells (Arunachalam and Doohan 2013), which in turn poses a serious health risk to consumers of food and feed produced with ingredients contaminated by these mycotoxins (Pestka and Smolinski 2005; Coppock and Jacobsen 2009). DON also acts as a virulence factor during the cereal infection process (Proctor et al. 1995; Jansen et al. 2005).

Currently, the majority of maize inbreds and hybrids are susceptible to ear rot, whereas wheat cultivars are, at most, moderately resistant to FHB (Mesterházy et al. 2012; Gilbert and Haber 2013). As a result, there is a pressing need to identify and analyze DON resistance mechanisms that can be applied *in planta*. A promising approach to achieve this involves the use of the yeast *Saccharomyces cerevisiae*, which has been identified as a model system to study the effects of trichothecene mycotoxins on eukaryotic cells (Doyle et al. 2009; Suzuki and Iwahashi 2012).

Plant orthologs of the plasma membrane ATP‐binding cassette (ABC) transporter Pdr5p of *S. cerevisiae* have the potential to be used as a first line of defense against DON and other trichothecene mycotoxins in crop plants. Pdr5p has been implicated as an exporter of DON and 15A‐DON (Suzuki and Iwahashi 2012; Mitterbauer and Adam, 2002). Furthermore, tobacco plants transformed with the *S. cerevisiae PDR5* gene demonstrated increased resistance to 4,15‐diacetoxyscirpenol, a trichothecene mycotoxin produced by *Fusarium poae* and *Fusarium equiseti* (Muhitch et al. 2000).

The pleiotropic drug resistance gene PDR5 from yeast was first described by Balzi et al. (1994). Since that time, the complete inventory of ABC proteins in yeast has been completed (Descottignies and Goffeau 1997). Pdr5p is the major ABC transporter in exponentially growing yeast, with a reported 42,000 molecules localized in the plasma membrane of each cell (Ghaemmaghami et al. 2003). Pdr5p couples the binding and hydrolysis of ATP with the export of over one hundred chemically and structurally distinct compounds, such as protein synthesis inhibitors, mycotoxins, anticancer drugs, and azole antifungals, from living cells (Higgins 1992; Kolaczkowski et al. 1996; Egner et al. 1998; Rees et al. 2009; Suzuki and Iwahashi 2012). Due to this remarkably broad range of substrate specificity, Pdr5p has become one of the most intensely studied ABC transporters.

Several inhibitors, including flavonoids, protein kinase C effectors, FK506, and enniatins, have been shown to specifically target the function of Pdr5p in yeast (Hiraga et al. 2005). *Fusarium avenaceum*, which commonly coinfects cereal grains with *F. graminearum* in Western Canada, has been shown to produce type A and B enniatins (Logrieco et al. 2002; Gräfenhan et al. 2013). A computational molecular model has been generated based on the crystal structures of resolved ABC transporters that share sequence homology with Pdr5p (Rutledge et al. 2011). According to this model, the transmembrane domains (TMDs) of Pdr5p, which ensure the unidirectional transport of substrates across the plasma membrane of yeast, form a large central cavity, or substrate‐binding pocket. Random and site‐directed mutagenesis, combined with phenotypic screening of the resulting Pdr5p mutants, suggested that the hydrophilic face of the substrate‐binding pocket contains at least seven different substrate‐binding sites (Egner et al. 2000; Tutulan‐Cunita et al. 2005). Two of these putative‐binding sites, located at residues S1360 and T1364, were of particular interest for this study, since single amino acid substitutions S1360A/F/T or T1364A/F/S had previously been shown to have a positive, negative, or neutral effect on both substrate specificity and inhibitor susceptibility of Pdr5p (Egner et al. 1998, 2000). Both S1360 and T1364 were therefore considered as potential residues involved in mediating Pdr5p resistance against *Fusarium* species.

In this study, we identified Pdr5p as the main exporter of the *F. graminearum* mycotoxins DON and 15A‐DON from yeast. We then generated a total of 38 mutants of Pdr5p, each containing an amino acid substitution at either residue S1360 or T1364. These mutants were individually expressed in yeast harboring a deletion of wild‐type (WT) Pdr5p and screened for their substrate specificity toward DON and 15A‐DON as well as their resistance to Pdr5p‐specific inhibitors FK506, enniatin B and *F. avenaceum* culture filtrate. Our results demonstrate that most of these Pdr5p variants maintained efficient export of both DON and 15A‐DON. Furthermore, specific mutants were more resistant than the WT to inhibition by FK506, enniatin B, or *F. avenaceum* culture filtrate, suggesting potential applications in plant resistance strategies.

## Experimental Procedures

### Chemicals and fungal metabolites

Working solutions of G418 (BioShop Canada Inc., Burlington, ON), FK506 (LC Laboratories, Woburn, MA), and enniatin B (Sigma‐Aldrich, St. Louis, MO) were prepared in 100% dimethyl sulfoxide (DMSO). DON and 15A‐DON were kindly provided by Dr. Barb Blackwell (ORDC, Ottawa, ON). *F. avenaceum* strain FaLH27 was isolated from wheat samples harvested in Nova Scotia in 2011 (Canadian Grain Commission, Winnipeg, MB) and deposited in the Canadian Collection of Fungal Cultures (AAFC, Ottawa, ON) with the strain designation DAOM242378. Using a two stage media protocol (modified from McCormick et al. 2004), six 250 mL flasks containing a glass microfiber filter (55 mm Whatman) and 50 mL first stage media per flask were inoculated with 2 × 10^6^ spores/mL of *F. avenaceum* FaLH27 and the fungi were grown at 28°C, 170 rpm for 6 days in the dark. The media were decanted, mycelia/filter rinsed with second stage media, and then resuspended in 50 mL second stage media and incubated at 28°C, 170 rpm for 12 days. Crude fungal filtrate (~300 mL) was fractionated on six 500 mg BondElut Plexa columns (Agilent Technologies, Mississauga, ON) and eluted with 5 mL of 100% MeOH. The six fractions were pooled, dried under vacuum, and resuspended at a concentration of 81.8 mg/mL in DMSO.

### Yeast strains and growth conditions

All *S. cerevisiae* yeast knockout (YKO) strains used in this study were derived from the haploid parental WT strain BY4741 (*Mat**a** his3∆1 leu2∆0 met 15∆0 ura3∆0*) and were purchased from Dharmacon (Lafayette, CO). The identity of each YKO strain was verified by PCR, using strain‐specific primers. The primer sequences and PCR product sizes for the YKO strains were obtained from the Saccharomyces Genome Deletion Project website (http://www-sequence.stanford.edu/group/yeast_deletion_project/downloads.html# instru). The WT and mutant *PDR5* yeast transformants used in this study are isogenic and were derived from the *∆pdr5* YKO strain. The identity of each transformant was verified by sequence analysis. Yeast strains were streaked on appropriate growth medium as follows: BY4741, on yeast peptone dextrose (YPD) agar; the YKO strains, on YPD agar with 200 *μ*g/mL G418; and the *∆pdr5* transformants, on synthetic dropout agar lacking uracil (SD‐Ura), and incubated at 30°C until individual colonies formed. Several colonies from each *PDR5* mutant transformant were restreaked onto appropriate fresh agar media and incubated at 30°C to obtain pure clonal isolates.

### Site‐directed mutagenesis of the *PDR5* gene

The WT *PDR5* gene was synthesized as a gene cassette containing the native *PDR5* promoter with an N‐terminus hemagglutinin (HA) epitope tag and the native *PDR5* 3’ untranslated region. The HA‐tagged *PDR5* was then cloned into the yeast expression vector p416CYC (DualSystems Biotech, Zurich, Switzerland) and sequence‐verified. All mutant *PDR5* genes encoding single amino acid substitutions at residue S1360 and T1364 were generated by in vitro site‐directed mutagenesis of the WT *PDR5* gene by GeneArt^®^ (Life Technologies, Regensburg, Germany). Cassettes containing each of the variant amino acid substitutions were cloned into the yeast expression vector p416CYC and sequence‐verified by GeneArt^®^. Each plasmid was then individually transformed into the *∆pdr5* YKO strain (Amberg et al. 2005).

### Yeast protein extract preparation and western blot analysis of Pdr5p

Whole‐cell protein extracts were prepared from subcultures of yeast, grown overnight (30°C, 300 rpm) in YPD broth to an optical density (OD; absorbance at 600 nm) of 3.5, using a protocol adapted from von der Haar (2007). A volume of 10^8^ cells was transferred to 2 mL screw‐capped tubes containing 75 *μ*L of 425–600 *μ*m acid‐washed glass beads (Sigma‐Aldrich). Cells were centrifuged (3800*g* for 5 min), and washed with sterile ice‐cold water; centrifuged again, and quick‐frozen in liquid nitrogen. Ice‐thawed pellets were suspended in 100 *μ*L lysis buffer [8 mol/L urea, 0.1 mol/L NaOH, 50 mmol/L EDTA, and 2% SDS, plus 20 *μ*L *β*‐mercaptoethanol and one cOmplete^TM^, Mini, EDTA‐free protease inhibitor cocktail tablet (Roche, Indianapolis, IN) per mL of lysis buffer], and cells were lysed in a Fast‐Prep Machine (Level 6 for 45 sec; MP Biomedicals, Santa Ana, CA). Lysates were incubated (55°C for 10 min), pH‐neutralized with 2.5 *μ*L of 4 mol/L acetic acid, and vortexed for 30 sec; lysates were incubated again (55°C for 10 min) and cleared by centrifugation (maximum speed for 5 min). The resulting protein extracts were transferred to sterile tubes and centrifuged (maximum speed for 2.5 min). Twenty‐five *μ*L of loading buffer was then added to the cleared supernatants, which were then stored at −80°C.

Proteins from aliquots of the supernatants were separated by SDS‐PAGE on 4–15% 26‐well Criterion^TM^ precast gels (Bio‐Rad, Mississauga, ON). Proteins were then blotted onto 0.2 *μ*m polyvinylidene difluoride (PVDF) membranes (Trans‐Blot^®^ Turbo^TM^ PVDF Transfer Packs [Bio‐Rad]) with a Trans‐Blot^®^ Turbo^TM^ Blotting System (Bio‐Rad), at 2.5A for 7 min. Blots were incubated in the following antibodies: polyclonal goat, anti‐Pdr5p antibody (yC‐18) (1:500 dilution; Santa Cruz Biotechnology, Santa Cruz, CA); monoclonal mouse, anti‐*β* actin antibody (1:1000 dilution; Abcam, Cambridge, UK); rabbit anti‐goat horseradish protein (HRP) antibody (1:120,000 dilution; Sigma‐Aldrich) and rabbit anti‐mouse HRP antibody (1:60,000 dilution; Jackson ImmunoReseach, West Grove, PA). Protein bands were detected by enhanced chemiluminescence (ECL; Clarity^TM^ Western ECL system; Bio‐Rad) and imaged on a ChemiDoc^TM^ XRS+ imaging system (Bio‐Rad).

### Microplate growth assays

Yeast cells were grown overnight (30°C, 300 rpm) in YPD; diluted to an OD of 0.1 in YPD and grown for 4 h (30°C, 300 rpm); then diluted again to an OD of 0.1 in YPD. In sterile Nunc^TM^ MicroWell^TM^ 96‐well plates (Thermo Scientific, Lafayette, CO), 50 *μ*L of cells was aliquoted to designated wells, and then 50 *μ*L of YPD containing 5% DMSO (negative control) or treatment was added to designated wells for a total of 100 *μ*L. The final percentage of DMSO was kept constant at 2.5% in all wells. The growth rate for each strain was recorded by absorbance at 600 nm, as measured by an Eon^TM^ Microplate Spectrophotometer (BioTek, Winooski, VT), using the Gen5^TM^ 2.0 data analysis software (BioTek). Area under the curve (AUC) was calculated by integration of the growth curves using Gen5^TM^. Relative growth ratios (%) were determined by dividing the AUC of treated cells by the AUC of control cells. Relative growth ratios were expressed as the mean, plus and minus the standard error of the mean (±SEM). Data were evaluated for equality of variance prior to statistical analysis. Data were then analyzed by one‐way analysis of variance (ANOVA), followed by Tukey's honestly significant difference test with significance accepted at *P* < 0.05. Statistical analysis was performed using the statistical software R (R Project).

## Results

### Pdr5p is the major exporter of DON and 15A‐DON

To assess whether additional members of the PDR network were also involved in *F. graminearum* mycotoxin export, PCR‐confirmed YKO strains lacking the individual genes responsible for expressing each ABC transporter with a known or putative role in the PDR network (Table S1) were phenotypically screened in the presence of DON and 15A‐DON. Yeast growth assays in liquid culture have been shown to be more accurate than assays on agar plates in detecting small toxin sensitivity variations among different yeast strains (Tutulan‐Cunita et al. 2005); therefore, all growth assays performed during this study were conducted in liquid culture.

**Figure 1:**
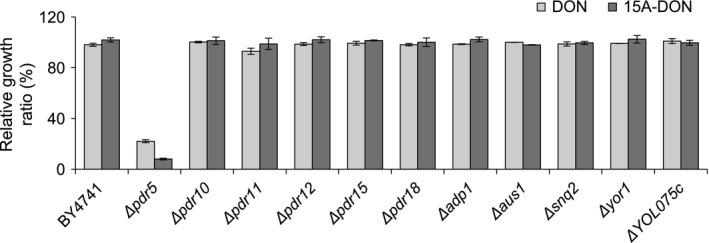
Relative growth ratio of yeast knockout strains in deoxynivalenol (DON) and 15A‐DON. Yeast cells were grown for 20 h in DON (125 *μ*g/mL) or 15A‐DON (25 *μ*g/mL). Relative growth ratios were determined as described in Experimental Procedures. All data are expressed as means ± standard error of the mean (SEM;* n* ≥ 3).

Aside from *∆pdr5*, whose growth was markedly reduced and almost completely inhibited in the presence of either DON or 15A‐DON, no significant growth defects or phenotypes were observed with the remaining ten transporter YKO strains, when compared with the WT (Fig. 1). Western blot analysis was performed to explicitly demonstrate that Pdr5p was not being expressed by *∆pdr5*. The anti‐Pdr5p antibodies detected a single band in the WT strain BY4741, which was used as a positive control for Pdr5p expression; however, this band was completely absent in *∆pdr5* (Fig. S1). Consistent with previous studies (Rutledge et al. 2011; Kueppers et al. 2013), this band was detected at approximately 150 kDa rather than the expected Pdr5p size of approximately 160 kDa.

### Single amino acid substitutions at residues S1360 and T1364 of Pdr5p

Amino acid residues S1360 and T1364 have been identified as being important in determining the ability of Pdr5p to mediate substrate specificity and to prevent inhibitor susceptibility (Egner et al. 2000). To our knowledge, aside from the S1360A/F/T and T1364A/F/S mutants (Egner et al. 1998, 2000), no analyses of additional mutants at either residue have previously been published. For this study, in vitro mutagenesis of the WT *PDR5* gene was performed to generate 19 *PDR5* S1360 variants, each with a different single amino acid substitution at residue S1360 of Pdr5p, and 19 *PDR5* T1364 variants, each with a different single amino acid substitution at residue T1364 of Pdr5p, for a total of 38 *PDR5* variants.

Since the presence of an N‐terminal HA‐tag did not affect the expression levels (Fig. S1) or function (Fig. S2) of WT Pdr5p, the 38 *PDR5* variants were each generated with an N‐terminal HA‐tag. Plasmids encoding each of the sequence‐verified *PDR5* gene variants were then individually transformed into the host strain *∆pdr5*. DNA sequence analysis of a segment of *PDR5* that included the region coding for amino acids S1360 and T1364 confirmed that each of the 38 *∆pdr5* transformants were expressing the correct *PDR5* variant (Figs. S3 and S4).

**Figure 2:**
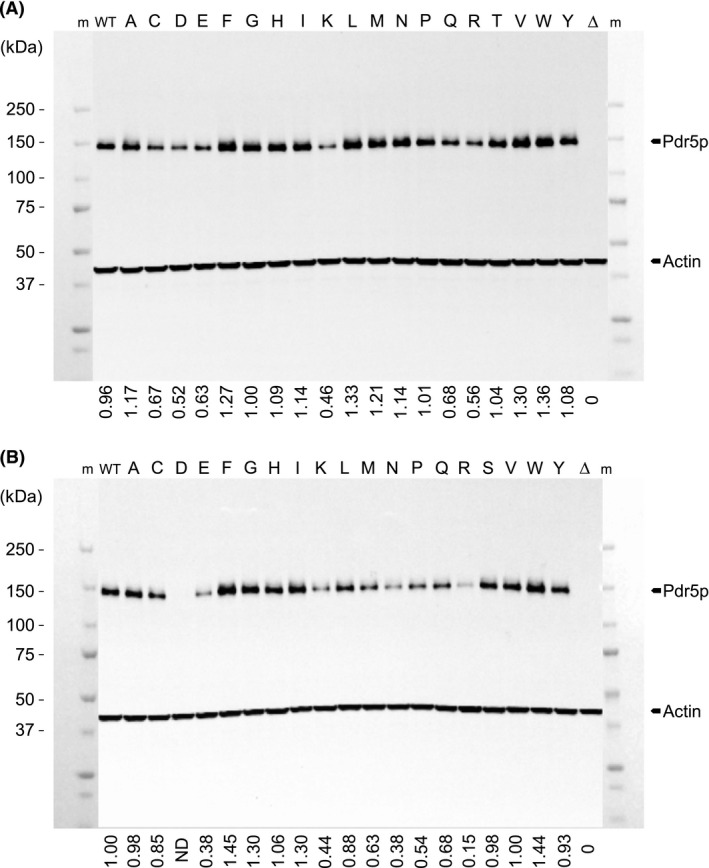
Expression levels of Pdr5p variants in yeast. (A) S1360 variants, (B) T1364 variants. Expression of the (A) S1360 Pdr5p variants and (B) T1364 Pdr5p variants in yeast relative to an actin control. Pdr5p was detected using an antibody directed against a C‐terminal peptide of Pdr5p (yC‐18). Actin was used as a loading control. Ratios of Pdr5p to actin are indicated below the panel. Lanes m: protein marker, Δ: empty plasmid control, WT: wild‐type Pdr5, and the variant amino acid is indicated above the panel. (*n *= 3).

Western blot analysis was used to detected a single band of approximately 150 kDa in all transformants (Fig. 2), which was completely absent in the empty plasmid control. Although a band is not visible on the blot shown in Figure 2 B, corresponding to the T1364D mutant, a single faint band of approximately 150 kDa was visible when the blot was overexposed. In all transformants, the relative quantity of actin was uniform and set at a value of 1.00 to enable the calculation of ratios of Pdr5p‐to‐actin for each transformant. Relative differences in Pdr5p expression level of each variant at both residues S1360 or T1364 were consistently observed in repeated western blots: expression was either higher than (e.g., ratios of 1.27–1.45), similar to, or less than [e.g., ratios of 0.88 to nondetected] that of the WT (Fig. 2).

### Most Pdr5p variants maintain substrate specificity for DON and 15A‐DON

**Figure 3:**
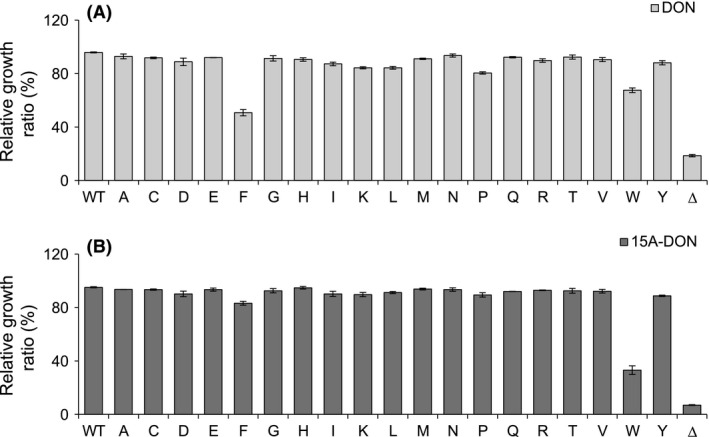
Relative growth ratios of S1360 Pdr5p variants in deoxynivalenol (DON) or 15A‐DON. Relative growth ratios of yeast strains expressing a S1360 Pdr5p variant, the wild‐type Pdr5p, and an empty plasmid control (Δ), grown for 20 h in either (A) DON (125 *μ*g/mL) or (B) 15A‐DON (25 *μ*g/mL). All data are expressed as means ± SEM (*n* ≥ 3).

**Figure 4:**
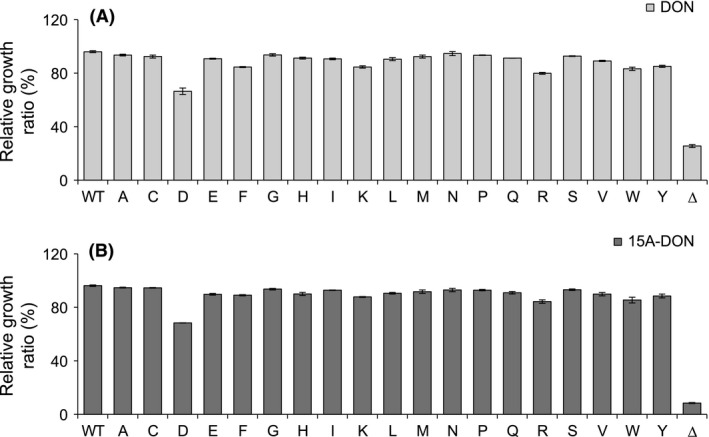
Relative growth ratios of T1364 Pdr5p variants in deoxynivalenol (DON) or 15A‐DON. Relative growth ratios of yeast strains expressing a T1364 Pdr5p variant the wild‐type Pdr5p, and an empty plasmid control (Δ), grown for 20 h in either (A) DON (125 *μ*g/mL) or (B) 15A‐DON (25 *μ*g/mL). All data are expressed as means ± SEM (*n* ≥ 3).

To determine the ability of each Pdr5p variant to export *F. graminearum* mycotoxins, assays were performed by growing each transformant in liquid culture with DON or 15A‐DON (Figs. 3 and 4). When compared to the transformant expressing the WT Pdr5p, growth of most of the Pdr5p mutant transformants was unaffected or marginally affected by DON or 15A‐DON. Aside from the S1360F, S1360W, and T1364D substitutions, all others maintained a high level of export of both trichothecenes when compared to the WT.

### Growth of the S1360 or T1364 variants in the presence of FK506 and trichothecenes

**Figure 5:**
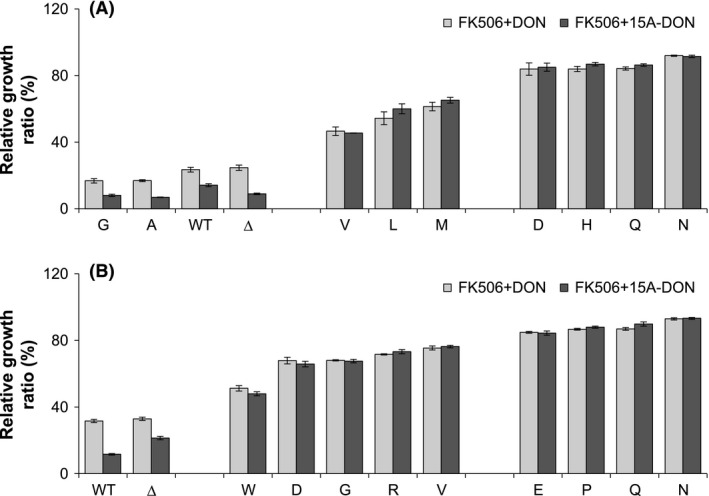
Relative growth ratios of selected (A) S1360 and (B) T1364 Pdr5p variants in FK506+ deoxynivalenol (DON) or FK506+15A‐DON. Relative growth ratios of yeast strains expressing either an S1360 or a T1364 Pdr5p variant (identified by the single‐letter code of the substituted amino acid), the wild‐type Pdr5p, and an empty plasmid control (Δ), grown for 20 h in either FK506+DON (light bars) or FK506+15A‐DON (dark bars). The ability of each strain to export either DON or 15A‐DON in the presence of FK506 varies from low (left) to high (right). All data are expressed as means ± SEM (*n* ≥ 3).

The main objective of this study was to identify optimal Pdr5p variants capable of maintaining a high level of DON and 15A‐DON export while demonstrating insensitivity to Pdr5p‐specific inhibitors. Growth of the S1360 or T1364 variants was compared in liquid culture with FK506 plus DON or FK506 plus 15A‐DON. FK506 alone did not affect the growth phenotypes of the variants (Tables S2 and S3) and the overall growth rates for each variant in the presence of FK506 were very similar in either mycotoxin (Fig. 5). Growth of the WT Pdr5p was drastically reduced in FK506 plus DON and FK506 plus 15A‐DON demonstrating the ability of FK506 to effectively inhibit the WT Pdr5p transporter. Although the growth of certain S1360 variants (e.g., S1360A/C/G/P) was compromised, many (e.g., S1360D/H/K/N/Q/Y) were capable of maintaining a high level of growth in DON or 15A‐DON in the presence of FK506. Furthermore, most T1364 variants, notably T1364E/N/P/Q, maintained a high level of growth in DON or 15A‐DON in the presence of FK506.

### Growth of the S1360 or T1364 variants in the presence of enniatin B and trichothecenes

**Figure 6:**
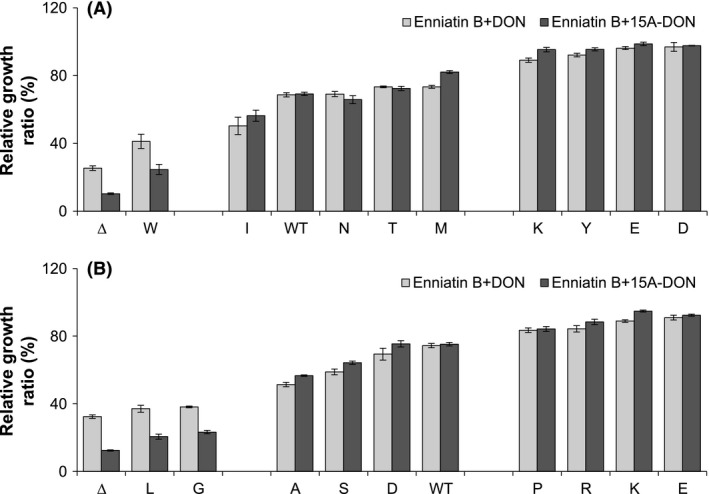
Relative growth ratios of selected S1360 (A) and T1364 (B) Pdr5p variants in enniatin B+ deoxynivalenol (DON) or enniatin B+15A‐DON. Relative growth ratios of selected yeast strains expressing an S1360 or T1364 Pdr5p variant (identified by the single‐letter code of the substituted amino acid), the wild‐type Pdr5p, and an empty plasmid control (Δ), grown for 20 h in either enniatin B+DON (light bars) or enniatin B+15A‐DON (dark bars). The ability of each strain to export either DON or 15A‐DON in the presence of enniatin B varies from low (left) to high (right). All data are expressed as means ± SEM (*n* ≥ 3).

Enniatin B is a known Pdr5p‐specific inhibitor produced by the cereal pathogen *F. avenaceum* (Hiraga et al. 2005) and it was, therefore, a biologically relevant inhibitor to analyze in this study. Growth assays were performed with each S1360 or T1364 variant in liquid culture with enniatin B plus DON or enniatin B plus 15A‐DON to determine their capacity to export DON or 15A‐DON in the presence of a known *Fusarium* Pdr5p‐specific inhibitor. Enniatin B alone did not affect the growth phenotypes of the variants (Table S2 and S3) and the overall growth rates for each variant in the presence of enniatin B were very similar in either mycotoxin (Fig. 6). Growth of certain variants, notably S1360W and T1364G/L, was compromised; however, other variants (e.g., S1360 D/E/K/Y and T1364E/K/P/R) were capable of maintaining a high level of growth in DON or 15A‐DON in the presence of enniatin B.

### Growth of selected Pdr5p variants in *F. avenaceum* filtrate

**Figure 7:**
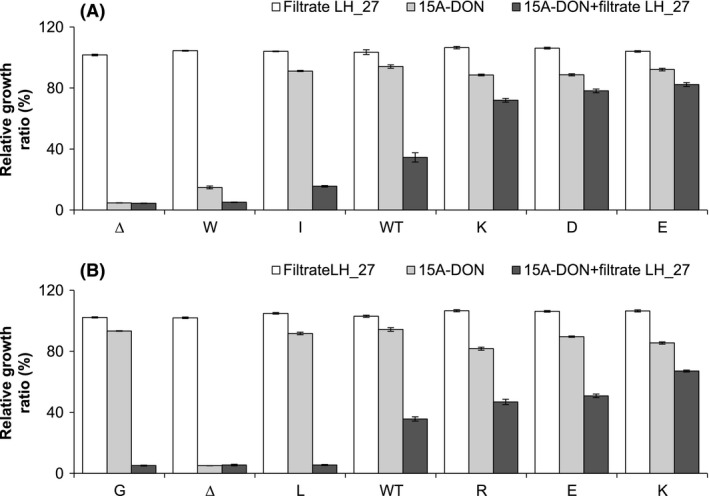
Relative growth ratios of selected Pdr5p S1360 (A) or T1364 (B) variants in *F. avenaceum* culture filtrate containing 15A‐ deoxynivalenol (DON). Relative growth ratios of selected yeast strains expressing an S1360 variant or a T1364 variant (identified by the single‐letter code of the substituted amino acid), the wild‐type Pdr5p, and an empty plasmid control (Δ), grown for 20 h in 15A‐DON (50 *μ*g/mL) (gray bars), *F. avenaceum* culture filtrate FaLH27 (0.5 *μ*L) (white bars), or *F. avenaceum* culture filtrate FaLH27 + 15A‐DON (0.5 *μ*L + 50 *μ*g/mL) (dark bars). All data are expressed as means ± SEM (*n* ≥ 3).

To further assess the ability of Pdr5p variants to enable yeast resistance to *F. avenaceum*, growth assays were performed with culture filtrate from *F. avenaceum* isolate FaLH27 (Fig. 7). The *F. avenaceum* FaLH27 filtrate contained all compounds produced and secreted by *F. avenaceum* grown in liquid culture that eluted with 100% MeOH from a Bond Elute Plexa column, including enniatins and other secondary metabolites. Since *F. avenaceum* does not produce trichothecene mycotoxins, 15A‐DON was added to the culture filtrate at 50 *μ*g/mL.

On its own, *F. avenaceum* FaLH27 culture filtrate did not significantly affect the growth of any of the Pdr5p variants that were tested (Fig. 7). Furthermore, the WT and all variants analyzed demonstrated a similar level of growth inhibition in the presence of 15A‐DON alone (at 50 *μ*g/mL), with the exception of S1360W. In *F. avenaceum* culture filtrate plus 15A‐DON, however, every Pdr5p variant demonstrated reduced growth at a level significantly greater than they had shown in 15A‐DON alone. Despite this, the level of this growth reduction was variable, and six of the 10 Pdr5p variants evaluated grew better than the WT. Certain variants (e.g., S1360D, S1360E, and T1364K) demonstrated minimal growth reduction in the presence of *F. avenaceum* culture filtrate plus 15A‐DON, whereas growth of the remaining variants was either substantially lower (e.g., T1364E and T1364R) or nearly completely inhibited (e.g., T1364G and T1364L).

## Discussion

A potential mechanism to generate *F. graminearum* resistance involves enhancing the ability to effectively export the trichothecene mycotoxins produced by this fungal pathogen. During crop infection by a 15A‐DON‐producing strain of *F. graminearum*, both DON and 15A‐DON typically accumulate within contaminated grain (Burlakoti et al. 2008; Gilbert et al. 2010). We observed that concentrations of 125 *μ*g/mL DON and 25 *μ*g/mL 15A‐DON were the maximum trichothecene concentrations that did not negatively affect growth of the WT yeast strain in liquid culture. The use of DON and 15A‐DON at these concentrations ensured that any growth inhibitory effects would be noticeable in the YKO strains corresponding to the transporters involved in exporting either of these mycotoxins. A concentration of DON five times greater than that of 15A‐DON was required to observe similar growth inhibitory effects in the WT strain which is in agreement with previous studies (Doyle et al. 2009; Suzuki and Iwahashi 2012).

While the role of Pdr5p in trichothecene tolerance has been discussed (Mitterbauer and Adam 2002; Suzuki and Iwahashi 2012), systematic testing of additional ABC transporters was not previously reported. In our study, all 11 known or putative transporters from the yeast PDR network were analyzed for their ability to export the *F. graminearum* mycotoxins DON and 15A‐DON (Table S1). Pdr5p and its functional homologs Snq2p and Yor1p are responsible for exporting distinct substrates but they have also been shown to possess overlapping substrate specificities (Kolaczkowski et al. 1998). Growth inhibitory assays performed using the haploid YKO strains of each of the 11 transporters demonstrated that aside from *∆pdr5*, the other 10 YKO strains grew at a similar rate compared to the WT in the presence of either mycotoxin. These results clearly showed that Pdr5p is the main yeast exporter of both DON and 15A‐DON and that the absence of this transporter leads to growth inhibition in the presence of these mycotoxins.

To be considered as candidates for mediating yeast resistance against a 15A‐DON‐producing strain of *F. graminearum*, Pdr5p mutants are required to not adversely affect yeast growth and to maintain export of both DON and 15A‐DON as efficiently as the WT Pdr5p. In the absence of mycotoxins, none of the S1360 or T1364 Pdr5p mutants affected yeast growth. Certain amino acid substitutions at residues S1360 or T1364 did have pronounced negative impacts on Pdr5p expression levels when compared to that of WT Pdr5p (Fig. 2). In accordance with previous results (Egner et al. 2000), the mutants S1360A/F/T and T1364A/F/S were expressed at levels similar to or slightly greater than that of WT Pdr5p.

While none of the Pdr5p S1360 or T1364 mutants were more effective than the WT Pdr5p at exporting either DON or 15A‐DON, all except for two S1360 mutants (Fig. 3) and all but one T1364 mutant (Fig. 4) were capable of maintaining a high level of export of both mycotoxins. Furthermore, despite the reduced protein expression levels of certain S1360 and T1364 Pdr5p mutants, all but T1364D demonstrated a high level of DON and 15A‐DON export. Pdr5p variant expression levels were not verified in yeast cells grown in the presence of DON or 15A‐DON; therefore, it is possible that expression was induced or reduced by either mycotoxin. Previous studies determined, however, that this was not the case for the Pdr5p WT, the S1360A/F/T or the T1364A/F/S mutants, which were expressed equally in the absence or presence of Pdr5p substrates (Egner et al. 2000).

It was previously shown that the S1360A and S1360T mutants were capable of maintaining drug export at a level comparable to that of WT Pdr5p, whereas drug export efficiency of S1360F was negatively affected (Egner et al. 2000). We also showed that S1360A and S1360T, but not S1360F, maintained a high level of DON and 15A‐DON export. The S1360F substitution had a negative effect on the ability of Pdr5p to transport both mycotoxins; however, it retained a significantly higher ability to export 15A‐DON. This was surprising, since 15A‐DON (approximately 340 Da) is a slightly larger molecule than DON (approximately 300 Da) and in this case, the relatively small side chain of serine (S) was replaced by the much larger aromatic side chain of phenylalanine (F). It has been proposed that substituting the hydrophilic amino acid serine with the hydrophobic amino acid phenylalanine at residue S1360, which is located on the hydrophilic side of the substrate‐binding pocket, influences its hydrophobic‐hydrophilic balance (Egner et al. 1998; Kueppers et al. 2013). Modification of this balance in the substrate‐binding pocket possibly caused a significant decrease in the affinity of Pdr5p to DON. Additionally, the S1360W substitution caused a significant reduction in Pdr5p transport efficiency of both DON and 15A‐DON. Like phenylalanine, tryptophan (W) has a large hydrophobic aromatic side chain, which was likely obstructing the export of both mycotoxins. Furthermore, 15A‐DON was transported less efficiently than DON, which could be due to its slightly larger molecular size. Since protein expression levels of the S1360F and S1360W mutants were greater than that of WT Pdr5p, growth reduction in the yeast strains expressing each of these mutants in either mycotoxin was not likely the result of decreased protein expression. Interestingly, the S1360Y mutation did not have a negative impact on the efficiency of Pdr5p to transport either DON or 15A‐DON. Like phenylalanine and tryptophan, tyrosine (Y) has a large hydrophobic aromatic side chain. Furthermore, the protein expression level of the S1360Y mutant was similar to that of WT Pdr5p.

The T1364D substitution had a significant negative effect on Pdr5p transport efficiency of both DON and 15A‐DON. Furthermore, protein expression levels of the T1364D mutant were exceptionally low. Misfolded Pdr5p mutants have been shown to be retained in the endoplasmic reticulum for degradation, preventing their normal localization at the plasma membrane. Yeast strains expressing Pdr5p mutants either with a C1427Y substitution or a L183P substitution were completely inhibited in the presence of all Pdr5p‐specific cytotoxic compounds tested, including azole antifungals (Egner et al. 1998; de Thozée et al. 2007). All S1360 and T1364 mutants analyzed in this study, however, maintained either an intermediate or high level of export of DON and 15A‐DON, which highly suggested that they all localized to the plasma membrane of yeast cells despite this not being verified experimentally. In the case of Pdr5p‐T1364D, the reason for intermediate rather than high level of mycotoxin export could be due to reduced affinity for both mycotoxins. A more likely explanation is that this mutation had a negative impact on Pdr5p expression and as a result, the yeast strain expressing this mutant had an insufficient quantity of expressed Pdr5p to maintain a suitable level of DON or 15A‐DON export. The level of export remained the same for both mycotoxins, suggesting that the side chain of aspartic acid (D) did not obstruct the passage of either compound. Furthermore, as opposed to the results at residue S1360, substitution of threonine at residue 1364 by amino acids with large hydrophobic side chains (T1364F/W/Y) did not negatively affect the ability of Pdr5p to transport either DON or 15A‐DON. This suggests that another factor, aside from amino acid size or shape, is important in determining substrate specificity by either S1360 or T1364. Single amino acid substitutions might be affecting the overall conformation of the substrate‐binding pocket.

The main objective of this study was to identify ideal Pdr5p variants capable of maintaining a high level of DON and 15A‐DON export while demonstrating insensitivity to Pdr5p‐specific inhibitors. FK506 (at 20 *μ*g/mL, 24.8 *μ*mol/L) was nontoxic to yeast cells (Tables S2 and S3). Growth inhibitory effects observed in yeast, when either trichothecene was combined with FK506, were therefore a result of FK506‐induced inhibition of mycotoxin transport. In agreement with results presented by Egner et al. (2000), S1360A showed very low growth ratios in DON and 15A‐DON, indicating hypersensitivity of this variant to FK506. Variants S1360F and S1360T demonstrated FK506 insensitivity, by maintaining an intermediate level of trichothecene export (Fig. 5). The reason for the intermediate rather than high level of mycotoxin export in the presence of FK506 was different for both mutants. The mutant S1360T maintained DON and 15A‐DON export as efficiently as the WT Pdr5p in the absence of FK506; however, this level of export was negatively affected by FK506. This was in contrast to S1360F, which was insensitive to FK506, but was unable to maintain mycotoxin export as efficiently as WT Pdr5p. Furthermore, relative to the growth inhibitory effects caused by DON or 15A‐DON alone, the combination of DON and FK506 had slightly smaller growth inhibitory effects than the combination of 15A‐DON and FK506 on yeast cells expressing S1360F (data not shown). This was likely the result of the higher level of toxicity of 15A‐DON on eukaryotic cells.

FK506 was previously shown to have no effect on expression of the WT Pdr5p, the S1360A/F/T mutants, or the T1364A/F/S mutants (Egner et al. 2000). In total, four T1364 and four S1360 mutants demonstrated insensitivity to FK506, by maintaining a high level of trichothecene export (Fig. 5). In agreement with results presented by Egner et al. (2000), the mutants T1364A/F/S demonstrated insensitivity toward FK506. Furthermore, similar to S1360F, the mutant T1364D was insensitive to FK506, despite its lack of mycotoxin transport efficiency. These mutants were, however, not among the most insensitive. In fact, ten of the T1364 mutants, of which six demonstrated reduced protein expression levels, maintained a high level of DON and 15A‐DON export in the presence of FK506. These mutants may have lost affinity for FK506 but this would need to be confirmed through kinetic studies.

Enniatin B is the major enniatin produced by *F. avenaceum*, a *Fusarium* species often associated with FHB‐affected cereals in Canada (Gräfenhan et al. 2013), and may synergistically increase the toxicity of other co‐occurring mycotoxins. Mutant ABC transporters demonstrating insensitivity toward enniatin B could therefore be considered as potential candidates for FHB resistance. Enniatin B (at 20 *μ*g/mL, 31.3 *μ*mol/L) was nontoxic to yeast cells (Table S2 and S3). Growth inhibitory effects observed in yeast in the presence of either trichothecene plus enniatin B were therefore a result of mycotoxin transport inhibition. Compared to FK506, which completely inhibited DON and 15A‐DON transport by the WT Pdr5p, enniatin B was not as potent (Figs. 5 and 6). This was contrary to results presented by Hiraga et al. (2005), who demonstrated that enniatin B was a more potent Pdr5p inhibitor than FK506. It must be taken into consideration that in our study, the concentrations of FK506 and enniatin B were about 5 times greater than those used by Hiraga et al. (2005). Pdr5p variant S1360A was slightly more sensitive to enniatin B combined with DON or 15A‐DON, whereas variant S1360F was insensitive, relative to WT Pdr5p, which agrees with results of Hiraga et al. (2005).

Substitution of serine with aspartic acid (D) or glutamic acid (E) at residue S1360 or substitution of threonine with glutamic acid (E) or lysine (K) at residue T1364 (Fig. 6) appears to reduce the affinity of Pdr5p for enniatin B. These mutants were therefore considered to be promising candidates for *F. avenaceum* resistance.

Under natural conditions, cereal grains are rarely contaminated by a single *Fusarium* species (Wagacha et al. 2012). In fact, grain sample analysis regularly detects multiple mycotoxins, including numerous trichothecenes, moniliformin (MON), and enniatins, produced by different *Fusarium* species (Gräfenhan et al. 2013; Tittlemier et al. 2013a,b). Selected yeast strains expressing Pdr5p mutants were grown to determine the ability of each mutant to maintain 15A‐DON export in the presence of *F. avenaceum* FaLH27 culture filtrate (Fig. 7). The culture filtrate used for these assays contained all nonaqueous compounds including enniatins, produced and secreted by the *F. avenaceum* isolate FaLH27 grown in liquid culture. The results obtained from these assays are encouraging, since Pdr5p mutants S1360D, S1360E, S1360K, and T1364K enabled significantly higher yeast growth in the presence of *F. avenaceum* culture filtrate plus 15A‐DON, when compared to the WT Pdr5p. Furthermore, protein expression levels of these four mutants were lower than that of the WT Pdr5p; therefore, these results were not caused by the overexpression of Pdr5p.

Using yeast as a model organism, this study has shown that the ABC transporter Pdr5p is the key exporter of the *F. graminearum* mycotoxin DON and its acetylated derivative 15A‐DON. This study has also demonstrated that the majority of single amino acid substitutions at residue S1360 or T1364 of Pdr5p are capable of maintaining DON and 15A‐DON export at a level similar to that of the WT Pdr5p. Furthermore, specific mutants have the ability to mediate insensitivity to the Pdr5p‐specific inhibitors FK506 and enniatin B while maintaining substrate specificity for DON and 15A‐DON. Mutants S1360D, S1360E, S1360K, and T1364K have been identified as candidates for resistance to enniatins. In addition to residues S1360 and T1364, molecular modeling and mutagenesis studies suggest that TMD2 of Pdr5p contains at least five other residues involved in substrate recognition. These five residues could also be mutagenized to analyze their ability to mediate inhibitor insensitivity and DON and 15A‐DON export.

Aside from T1364D, all other Pdr5p mutants that demonstrated reduced protein expression levels were capable of maintaining a level of DON and 15A‐DON export similar to that of WT Pdr5p. These results are encouraging and by increasing the expression of any of these mutants it might be possible to improve their mycotoxin export efficiency. The yeast *PDR1‐3* mutation, which causes the overexpression of genes controlled by the PDR transcription factor PDR1, might allow for the overexpression of these Pdr5p variants. This would be particularly interesting to perform with the Pdr5p variant T1364D. While this was the only mutant that demonstrated both reduced levels of protein expression and DON and 15A‐DON export, it also remained completely insensitive to inhibition by either FK506 or enniatin B.

Since crop infections are rarely caused by a single *Fusarium* species, it would be useful to identify Pdr5p mutants that could be considered as candidates for resistance to other *Fusarium* species. Furthermore, it might be possible to generate a Pdr5p mutant with substitutions at multiple key residues, enabling resistance to multiple species of *Fusarium*. In this study, preliminary results demonstrated that the S1360D, S1360E, S1360K, and T1364K mutants were capable of mediating yeast resistance to the *F. avenaceum* culture filtrate while maintaining the capacity to export trichothecenes.

Ultimately, knowledge gained through the use of yeast may be extended to plants, in an attempt to improve their ability to export mycotoxins, while mediating insensitivity to inhibitory compounds produced by *Fusarium* species. It was previously demonstrated that transgenic tobacco seedlings expressing *S. cerevisiae* Pdr5p displayed a significant increase in resistance to the trichothecene mycotoxin 4,15‐diacetoxyscirpenol (Muhitch et al. 2000).

Future research could involve modifying Pdr5p‐like plasma membrane transporters found in plants to confer multiple mycotoxin resistance. Since these transporters have not yet been extensively studied, it is unclear which are involved in mycotoxin export. In *A. thaliana* and *Zea mays*, 15 and 17 Pdr5p‐like proteins have been identified, respectively (van den Brûle and Smart 2002; Pang et al. 2013).

Considering the economic repercussions and health risks associated with the contamination of cereal grains by DON and its acetylated derivatives, it is vital to identify effective resistance mechanisms against them. The findings of this study strongly suggest that the transporter Pdr5p could be engineered to mediate yeast resistance to *F. avenaceum*,* F. graminearum*, as well as other *Fusarium* species. If this knowledge could then be applied to crop plants, it could have important implications in ensuring the safety of the food and feed supply chains.

## Conflict of interest

None declared.

## Supporting information


**Figure S1.** Expression levels of HA‐tagged and nontagged wild‐type Pdr5p in yeast.

**Figure S2.** Relative growth ratios of HA‐tagged and nontagged wild‐type Pdr5p in yeast.

**Figure S3.** Portion of the aligned amino acid sequences obtained following the sequencing of each S1360 Pdr5p variant.

**Figure S4.** Portion of the aligned amino acid sequences obtained following the sequencing of each T1364 Pdr5p variant.

**Table S1.** *Saccharomyces cerevisiae* ABC transporters with known or putative roles in the PDR network.

**Table S2.** Relative growth ratios of S1360 Pdr5p in FK506 or enniatin B.

**Table S3.** Relative growth ratios of T1364 Pdr5p in FK506 or enniatin B.

## References

[mbo3381-bib-0001] Amberg, D. C. , D. J. Burke , and J. N. Strathern . 2005 High‐efficiency transformation of yeast Pp. 109–111 *in* Amberg D. C., Burke D. J. and Strathern J. N., eds. Methods in yeast genetics: a cold spring harbor laboratory course manual. Cold Spring Harbor Laboratory Press, Cold Spring Harbor, NY.

[mbo3381-bib-0002] Arunachalam, C. , and F. M. Doohan . 2013 Trichothecene toxicity in eukaryotes: cellular and molecular mechanisms in plants and animals. Toxicol. Lett. 217:149–158. 23274714 10.1016/j.toxlet.2012.12.003

[mbo3381-bib-0003] Balzi, E. , M. Wang , S. Leterme , L. Van Dyck , and A. Goffeau . 1994 PDR5, a novel yeast multidrug resistance conferring transporter controlled by the transcription regulator PDR1. J. Biol. Chem. 269:2206–2214. 8294477

[mbo3381-bib-0004] van den Brûle, S. , and C. C. Smart . 2002 The plant PDR family of ABC transporters. Planta 216:95–106. 12430018 10.1007/s00425-002-0889-z

[mbo3381-bib-0005] Burlakoti, R. , S. Ali , G. Secor , S. Neate , M. McMullen , and T. Adhikari . 2008 Comparative mycotoxin profiles of Gibberella zeae populations from barley, wheat, potatoes, and sugar beets. Appl. Environ. Microbiol. 74:6513–6520. 18791024 10.1128/AEM.01580-08 PMC2576685

[mbo3381-bib-0006] Coppock, R. W. , and B. J. Jacobsen . 2009 Mycotoxins in animal and human patients. Toxicol. Ind. Health 25:637–655. 19793772 10.1177/0748233709348263

[mbo3381-bib-0007] Descottignies, A. , and A. Goffeau . 1997 Complete inventory of the yeast ABC proteins. Nat. Genet. 15:137–145. 9020838 10.1038/ng0297-137

[mbo3381-bib-0008] Doyle, P. J. , H. Saeed , A. Hermans , S. C. Gleddie , and G. Hussack . 2009 Intracellular expression of a single domain antibody reduces cytotoxicity of 15‐acetyldeoxynivalenol in yeast. J. Biol. Chem. 284:35029–35039. 19783651 10.1074/jbc.M109.045047 PMC2787364

[mbo3381-bib-0009] Egner, R. , F. E. Rosenthal , A. Kralli , D. Sanglard , and K. Kuchler . 1998 Genetic separation of FK506 susceptibility and drug transport in the yeast Pdr5 ATP‐binding cassette multidrug resistance transporter. Mol. Biol. Cell 9:523–543. 9450972 10.1091/mbc.9.2.523 PMC25282

[mbo3381-bib-0010] Egner, R. , B. E. Bauer , and K. Kuchler . 2000 The transmembrane domain 10 of the yeast Pdr5p ABC antifungal efflux pump determines both substrate specificity and inhibitor susceptibility. Mol. Microbiol. 35:1255–1263. 10712705 10.1046/j.1365-2958.2000.01798.x

[mbo3381-bib-0011] Ghaemmaghami, S. , W. K. Huh , K. Bower , R. W. Howson , A. Belle , N. Dephoure , et al. 2003 Global analysis of protein expression in yeast. Nature 425:737–741. 14562106 10.1038/nature02046

[mbo3381-bib-0012] Gilbert, J. , and S. Haber . 2013 Overview of some recent research developments in fusarium head blight of wheat. Can. J. Plant Pathol. 35:149–174.

[mbo3381-bib-0013] Gilbert, J. , R. M. Clear , T. J. Ward , D. Gaba , A. Tekauz , T. Turkington , et al. 2010 Relative aggressiveness and production of 3‐ or 15‐acetyl deoxynivalenol and deoxynivalenol by *Fusarium graminearum* in spring wheat. Can. J. Plant Pathol. 32:146–152.

[mbo3381-bib-0014] Gräfenhan, T. , S. K. Patrick , M. Roscoe , R. Trelka , D. Gaba , J. M. Chan , et al. 2013 Fusarium damage incereal grains from Western Canada. 1. Phylogenetic analysis of moniliformin‐producing fusarium species and their natural occurrence in mycotoxin‐contaminated wheat, oats, and rye. J. Agric. Food Chem. 61:5425–5437. 23683177 10.1021/jf400651p

[mbo3381-bib-0015] von der Haar, T. 2007 Optimized protein extraction for quantitative proteomics of yeasts. PLoS One 2:e1078. 17957260 10.1371/journal.pone.0001078 PMC2031916

[mbo3381-bib-0016] Higgins, C. F. 1992 ABC transporters: from microorganisms to man. Annu. Rev. Cell Biol. 8:67–113. 1282354 10.1146/annurev.cb.08.110192.000435

[mbo3381-bib-0017] Hiraga, K. , S. Yamamoto , H. Fukuda , N. Hamanaka , and K. Oda . 2005 Enniatin has a new function as an inhibitor of Pdr5p, one of the ABC transporters in *Saccharomyces cerevisiae* . Biochem. Biophys. Res. Commun. 328:1119–1125. 15707993 10.1016/j.bbrc.2005.01.075

[mbo3381-bib-0018] Jansen, C. , D. von Wettstein , W. Schäfer , K. H. Kogel , A. Felk , and F. J. Maier , et al. 2005 Infection patterns in barley and wheat spikes inoculated with wild‐type and trichodiene synthase gene disrupted *Fusarium graminearum* . Proc. Natl. Acad. Sci. USA 102:16892–16897. 16263921 10.1073/pnas.0508467102 PMC1283850

[mbo3381-bib-0019] Kolaczkowski, M. , M. van der Rest , A. Cybularz‐Kolaczkowska , J. P. Soumillion , W. N. Konings , and A. Goffeau . 1996 Anticancer drugs, ionophoric peptides, and steroids as substrates of the yeast multidrug transporter Pdr5p. J. Biol. Chem. 271:31543–31548. 8940170 10.1074/jbc.271.49.31543

[mbo3381-bib-0020] Kolaczkowski, M. , A. Kolaczowska , J. Luczynski , S. Witek , and A. Goffeau . 1998 In vivo characterization of the drug resistance profile of the major ABC transporters and other components of the yeast pleiotropic drug resistance network. Microb. Drug Resist. 4:143–158. 9818966 10.1089/mdr.1998.4.143

[mbo3381-bib-0021] Kueppers, P. , R. P. Gupta , J. Stindt , S. H. Smits , and L. Schmitt . 2013 Functional impact of a single mutation within the transmembrane domain of the multidrug ABC transporter Pdr5. Biochemistry 52:2184–2195. 23464591 10.1021/bi3015778

[mbo3381-bib-0022] Logrieco, A. , A. Rizzo , R. Ferracane , and A. Ritieni . 2002 Occurrence of beauvericin and enniatins in wheat affected by *Fusarium avenaceum* head blight. Appl. Environ. Microbiol. 68:82–85. 11772612 10.1128/AEM.68.1.82-85.2002 PMC126553

[mbo3381-bib-0023] McCormick, S. P. , L. J. Harris , N. J. Alexander , T. Ouellet , A. Saparno , S. Allard , et al. 2004 *Tri1* in *Fusarium graminearum* encodes a P450 oxygenase. Appl. Environ. Microbiol. 70:2044–2051. 15066795 10.1128/AEM.70.4.2044-2051.2004 PMC383062

[mbo3381-bib-0024] McMullen, M. , G. Bergstrom , E. De Wolf , R. Dill‐Macky , D. Hershman , D. Shaner , et al. 2012 A united effort to fight an enemy of wheat and barley: fusarium head blight. Plant Dis. 96:1712–1728. 10.1094/PDIS-03-12-0291-FE 30727259

[mbo3381-bib-0025] Mesterházy, A. , M. Lemmens , and L. M. Reid . 2012 Breeding for resistance to ear rots caused by *Fusarium* spp. in maize – a review. Plant Breed. 131:1–19.

[mbo3381-bib-0026] Mitterbauer, R. , and G. Adam . 2002 *Saccharomyces cerevisiae* and *Arabidopsis thaliana*: useful model systems for the identification of molecular mechanisms involved in resistance of plants to toxins. Eur. J. Plant Pathol. 108:699–703.

[mbo3381-bib-0027] Muhitch, M. J. , S. P. McCormick , N. J. Alexander , and T. M. Hohn . 2000 Transgenic expression of the TRI101 or PDR5 gene increases resistance of tobacco to the phytotoxic effects of the trichothecene 4,15‐diacetoxyscirpenol. Plant Sci. 157:201–207. 10960733 10.1016/s0168-9452(00)00282-x

[mbo3381-bib-0028] Pang, K. , Y. Li , M. Liu , Z. Meng , and Y. Yu . 2013 Inventory and general analysis of the ATP‐binding cassette (ABC) gene superfamily in maize (*Zea mays* L.). Gene 526:411–428. 23747399 10.1016/j.gene.2013.05.051

[mbo3381-bib-0029] Pestka, J. J. , and A. T. Smolinski . 2005 Deoxynivalenol: toxicology and potential effects on humans. J. Toxicol. Environ. Health B Crit. Rev. 8:39–69. 15762554 10.1080/10937400590889458

[mbo3381-bib-0030] Proctor, R. H. , T. M. Hohn , and S. McCormick . 1995 Reduced virulence of *Gibberella zeae* caused by disruption of a trichothecene toxin biosynthetic gene. Mol. Plant Microbe Interact. 8:593–601. 8589414 10.1094/mpmi-8-0593

[mbo3381-bib-0031] Rees, D. C. , E. Johnson , and O. Lewinson . 2009 ABC transporters: the power to change. Nat. Rev. Mol. Cell Biol. 10:218–227. 19234479 10.1038/nrm2646 PMC2830722

[mbo3381-bib-0032] Rutledge, R. M. , L. Esser , J. Ma , and D. Xia . 2011 Toward understanding the mechanism of action of the yeast multidrug resistance transporter Pdr5p: a molecular modeling study. J. Struct. Biol. 173:333–344. 21034832 10.1016/j.jsb.2010.10.012 PMC3026082

[mbo3381-bib-0033] Suzuki, T. , and Y. Iwahashi . 2012 Comprehensive gene expression analysis of type B trichothecenes. J. Agric. Food Chem. 60:9519–9527. 22897823 10.1021/jf3020975

[mbo3381-bib-0034] de Thozée, C. P. , S. Cronin , A. Goj , J. Golin , and M. Ghislain . 2007 Subcellular trafficking of the yeast plasma membrane ABC transporter, Pdr5, is impaired by a mutation in the N‐terminal nucleotide‐binding fold. Mol. Microbiol. 63:811–825. 17302805 10.1111/j.1365-2958.2006.05562.x

[mbo3381-bib-0035] Tittlemier, S. A. , M. Roscoe , R. Trelka , D. Gaba , J. M. Chan , S. K. Patrick , et al. 2013a Fusarium damage in small cereal grains from Western Canada. 2. Occurrence of *Fusarium* toxins and their source organisms in durum wheat harvested in 2010. J. Agric. Food Chem. 61:5438–5448. 23683132 10.1021/jf400652e

[mbo3381-bib-0036] Tittlemier, S. A. , D. Gaba , and J. M. Chan . 2013b Monitoring of *Fusarium* trichothecenes in Canadian cereal grain shipments from 2010 to 2012. J. Agric. Food Chem. 61:7412–7418. 23844863 10.1021/jf4019257

[mbo3381-bib-0037] Tutulan‐Cunita, A. C. , M. Mikoshi , M. Mizunuma , D. Hirata , and T. Miyakawa . 2005 Mutational analysis of the yeast multidrug resistance ABC transporter Pdr5p with altered drug specificity. Genes Cells 10:409–420. 15836770 10.1111/j.1365-2443.2005.00847.x

[mbo3381-bib-0038] Wagacha, J. M. , E. C. Oerke , H. W. Dehne , and U. Steiner . 2012 Interactions of *Fusarium* species during prepenetration development. Fungal Biol. 116:836–847. 22749170 10.1016/j.funbio.2012.05.001

