# Supplementary figures and images for: Protein engineering of Saccharomyces cerevisiae transporter Pdr5p identifies key residues that impact Fusarium mycotoxin export and resistance to inhibition
Source: Microbiologyopen. 2016 Jun 4;5(6):979–91. doi: 10.1002/mbo3.381 (PMC5221463; doi:10.1002/mbo3.381)

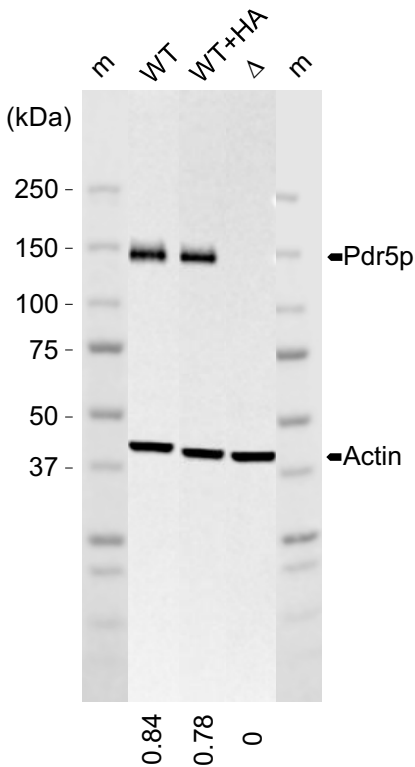

Supplement: Supplementary file 1 — Figure S1. Expression levels of HA‐tagged and nontagged wild‐type Pdr5p in yeast. [file MBO3-5-979-s001.pdf]

**A**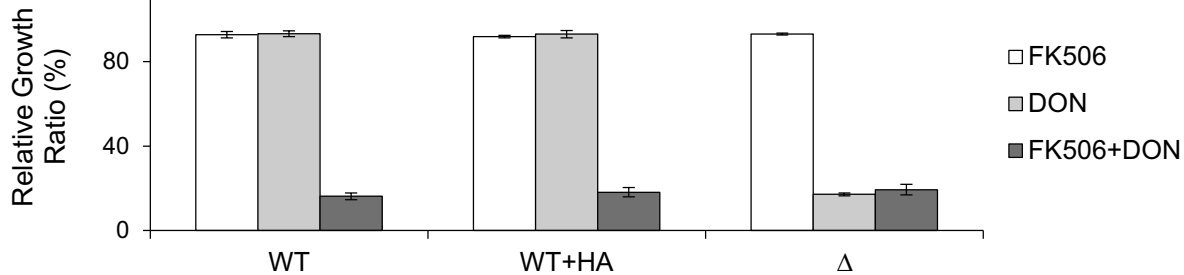**B**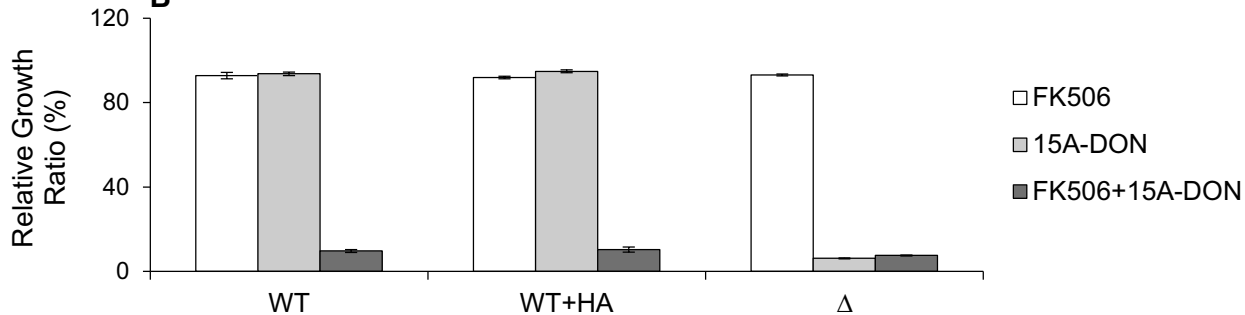

Supplement: Supplementary file 2 — Figure S2. Relative growth ratios of HA‐tagged and nontagged wild‐type Pdr5p in yeast. [file MBO3-5-979-s002.pdf]
