# Supplementary material for: Protein engineering of Saccharomyces cerevisiae transporter Pdr5p identifies key residues that impact Fusarium mycotoxin export and resistance to inhibition
Source: Microbiologyopen. 2016 Jun 4;5(6):979–91. doi: 10.1002/mbo3.381 (PMC5221463; doi:10.1002/mbo3.381)
Supplement: Supplementary file 3 — Figure S3. Portion of the aligned amino acid sequences obtained following the sequencing of each S1360 Pdr5p variant. [file MBO3-5-979-s003.pdf]

TMH11 (1355-1379)

```
S1360A-1_protein  GSMGLLVI SFNQVAESAANLAALLFTMSLSFCGVMTTPSAMPR
S1360A-4_protein  GSMGLLVI SFNQVAESAANLAALLFTMSLSFCGVMTTPSAMPR
S1360C-1_protien  GSMGLLVI SFNQVAESAANLACLLFTMSLSFCGVMTTPSAMPR
S1360C-3_protein  GSMGLLVI SFNQVAESAANLACLLFTMSLSFCGVMTTPSAMPR
S1360D-5_protein  GSMGLLVI SFNQVAESAANLADLLFTMSLSFCGVMTTPSAMPR
S1360D-6_protein  GSMGLLVI SFNQVAESAANLADLLFTMSLSFCGVMTTPSAMPR
S1360E-1_protein  GSMGLLVI SFNQVAESAANLAELLFTMSLSFCGVMTTPSAMPR
S1360E-3_protein  GSMGLLVI SFNQVAESAANLAELLFTMSLSFCGVMTTPSAMPR
S1360F-1_protein  GSMGLLVI SFNQVAESAANLAFLLFTMSLSFCGVMTTPSAMPR
S1360F-4_protein  GSMGLLVI SFNQVAESAANLAFLLFTMSLSFCGVMTTPSAMPR
S1360G-3_protein  GSMGLLVI SFNQVAESAANLAGLLFTMSLSFCGVMTTPSAMPR
S1360G-4_protein  GSMGLLVI SFNQVAESAANLAGLLFTMSLSFCGVMTTPSAMPR
S1360H-1_protein  GSMGLLVI SFNQVAESAANLAHLLFTMSLSFCGVMTTPSAMPR
S1360H-5_protein  GSMGLLVI SFNQVAESAANLAHLLFTMSLSFCGVMTTPSAMPR
S1360I-3_protein  GSMGLLVI SFNQVAESAANLAI LLFTMSLSFCGVMTTPSAMPR
S1360I-4_protein  GSMGLLVI SFNQVAESAANLAI LLFTMSLSFCGVMTTPSAMPR
S1360K-1_protein  GSMGLLVI SFNQVAESAANLAKLLFTMSLSFCGVMTTPSAMPR
S1360K-4_protein  GSMGLLVI SFNQVAESAANLAKLLFTMSLSFCGVMTTPSAMPR
S1360L-1_protein  GSMGLLVI SFNQVAESAANLALLLFTMSLSFCGVMTTPSAMPR
S1360L-3_protein  GSMGLLVI SFNQVAESAANLALLLFTMSLSFCGVMTTPSAMPR
S1360M-4_protein  GSMGLLVI SFNQVAESAANLAMLLFTMSLSFCGVMTTPSAMPR
S1360M-5_protein  GSMGLLVI SFNQVAESAANLAMLLFTMSLSFCGVMTTPSAMPR
S1360N-2_protein  GSMGLLVI SFNQVAESAANLANLLFTMSLSFCGVMTTPSAMPR
S1360N-4_protein  GSMGLLVI SFNQVAESAANLANLLFTMSLSFCGVMTTPSAMPR
S1360P-1_protein  GSMGLLVI SFNQVAESAANLAPLLFTMSLSFCGVMTTPSAMPR
S1360P-3_protein  GSMGLLVI SFNQVAESAANLAPLLFTMSLSFCGVMTTPSAMPR
S1360Q-4_protein  GSMGLLVI SFNQVAESAANLAQLLFTMSLSFCGVMTTPSAMPR
S1360Q-7_protein  GSMGLLVI SFNQVAESAANLAQLLFTMSLSFCGVMTTPSAMPR
S1360R-1_protein  GSMGLLVI SFNQVAESAANLARLLFTMSLSFCGVMTTPSAMPR
S1360R-4_protein  GSMGLLVI SFNQVAESAANLARLLFTMSLSFCGVMTTPSAMPR
S1360T-3_protein  GSMGLLVI SFNQVAESAANLATLLFTMSLSFCGVMTTPSAMPR
S1360T-4_protein  GSMGLLVI SFNQVAESAANLATLLFTMSLSFCGVMTTPSAMPR
S1360V-2_protein  GSMGLLVI SFNQVAESAANLAVLLFTMSLSFCGVMTTPSAMPR
S1360V-4_protein  GSMGLLVI SFNQVAESAANLAVLLFTMSLSFCGVMTTPSAMPR
S1360W-2_protein  GSMGLLVI SFNQVAESAANLAWLLFTMSLSFCGVMTTPSAMPR
S1360W-4_protein  GSMGLLVI SFNQVAESAANLAWLLFTMSLSFCGVMTTPSAMPR
S1360Y-1_protein  GSMGLLVI SFNQVAESAANLAYLLFTMSLSFCGVMTTPSAMPR
S1360Y-5_protein  GSMGLLVI SFNQVAESAANLAYLLFTMSLSFCGVMTTPSAMPR
```
