# Supplementary material for: Protein engineering of Saccharomyces cerevisiae transporter Pdr5p identifies key residues that impact Fusarium mycotoxin export and resistance to inhibition
Source: Microbiologyopen. 2016 Jun 4;5(6):979–91. doi: 10.1002/mbo3.381 (PMC5221463; doi:10.1002/mbo3.381)
Supplement: Supplementary file 4 — Figure S4. Portion of the aligned amino acid sequences obtained following the sequencing of each T1364 Pdr5p variant. [file MBO3-5-979-s004.pdf]

TMH11 (1355-1379)

```
T1364A-1_protein  GSMGLLVI SFNQVAESAANLASLLFAMSLSFCGVMTTPSAMPR
T1364A-2_protein  GSMGLLVI SFNQVAESAANLASLLFAMSLSFCGVMTTPSAMPR
T1364C-1_protein  GSMGLLVI SFNQVAESAANLASLLFCMSLSFCGVMTTPSAMPR
T1364C-4_protein  GSMGLLVI SFNQVAESAANLASLLFCMSLSFCGVMTTPSAMPR
T1364D-1_protein  GSMGLLVI SFNQVAESAANLASLLFDMSLSFCGVMTTPSAMPR
T1364D-4_protein  GSMGLLVI SFNQVAESAANLASLLFDMSLSFCGVMTTPSAMPR
T1364E-1_protein  GSMGLLVI SFNQVAESAANLASLLFEMSLSFCGVMTTPSAMPR
T1364E-2_protein  GSMGLLVI SFNQVAESAANLASLLFEMSLSFCGVMTTPSAMPR
T1364F-1_protein  GSMGLLVI SFNQVAESAANLASLLFFMSLSFCGVMTTPSAMPR
T1364F-3_protein  GSMGLLVI SFNQVAESAANLASLLFFMSLSFCGVMTTPSAMPR
T1364G-5_protein  GSMGLLVI SFNQVAESAANLASLLFGMSLSFCGVMTTPSAMPR
T1364G-6_protein  GSMGLLVI SFNQVAESAANLASLLFGMSLSFCGVMTTPSAMPR
T1364H-2_protein  GSMGLLVI SFNQVAESAANLASLLFHMSLSFCGVMTTPSAMPR
T1364H-5_protein  GSMGLLVI SFNQVAESAANLASLLFHMSLSFCGVMTTPSAMPR
T1364I-2_protein  GSMGLLVI SFNQVAESAANLASLLFIMSLSFCGVMTTPSAMPR
T1364I-3_protein  GSMGLLVI SFNQVAESAANLASLLFIMSLSFCGVMTTPSAMPR
T1364K-1_protein  GSMGLLVI SFNQVAESAANLASLLFKMSLSFCGVMTTPSAMPR
T1364K-2_protein  GSMGLLVI SFNQVAESAANLASLLFKMSLSFCGVMTTPSAMPR
T1364L-1_protein  GSMGLLVI SFNQVAESAANLASLLFLMSLSFCGVMTTPSAMPR
T1364L-5_protein  GSMGLLVI SFNQVAESAANLASLLFLMSLSFCGVMTTPSAMPR
T1364M-1_protein  GSMGLLVI SFNQVAESAANLASLLFMMSLSFCGVMTTPSAMPR
T1364M-4_protein  GSMGLLVI SFNQVAESAANLASLLFMMSLSFCGVMTTPSAMPR
T1364N-2_protein  GSMGLLVI SFNQVAESAANLASLLFNMSLSFCGVMTTPSAMPR
T1364N-3_protein  GSMGLLVI SFNQVAESAANLASLLFNMSLSFCGVMTTPSAMPR
T1364P-3_protein  GSMGLLVI SFNQVAESAANLASLLFPMSLSFCGVMTTPSAMPR
T1364P-4_protein  GSMGLLVI SFNQVAESAANLASLLFPMSLSFCGVMTTPSAMPR
T1364Q-4_protein  GSMGLLVI SFNQVAESAANLASLLFQMSLSFCGVMTTPSAMPR
T1364Q-6_protein  GSMGLLVI SFNQVAESAANLASLLFQMSLSFCGVMTTPSAMPR
T1364R-1_protein  GSMGLLVI SFNQVAESAANLASLLFRMSLSFCGVMTTPSAMPR
T1364R-6_protein  GSMGLLVI SFNQVAESAANLASLLFRMSLSFCGVMTTPSAMPR
T1364S-2_protein  GSMGLLVI SFNQVAESAANLASLLFSMSLSFCGVMTTPSAMPR
T1364S-4_protein  GSMGLLVI SFNQVAESAANLASLLFSMSLSFCGVMTTPSAMPR
T1364V-1_protein  GSMGLLVI SFNQVAESAANLASLLFVMSLSFCGVMTTPSAMPR
T1364V-3_protein  GSMGLLVI SFNQVAESAANLASLLFVMSLSFCGVMTTPSAMPR
T1364W-4_protein  GSMGLLVI SFNQVAESAANLASLLFWMSLSFCGVMTTPSAMPR
T1364W-5_protein  GSMGLLVI SFNQVAESAANLASLLFWMSLSFCGVMTTPSAMPR
T1364Y-2_protein  GSMGLLVI SFNQVAESAANLASLLFYMSLSFCGVMTTPSAMPR
T1364Y-6_protein  GSMGLLVI SFNQVAESAANLASLLFYMSLSFCGVMTTPSAMPR
```
