# Supplementary material for: Protein engineering of Saccharomyces cerevisiae transporter Pdr5p identifies key residues that impact Fusarium mycotoxin export and resistance to inhibition
Source: Microbiologyopen. 2016 Jun 4;5(6):979–91. doi: 10.1002/mbo3.381 (PMC5221463; doi:10.1002/mbo3.381)
Supplement: Supplementary file 5 — Table S1. Saccharomyces cerevisiae ABC transporters with known or putative roles in the PDR network. [file MBO3-5-979-s005.docx]

| **Standard Name^a^** | **Systematic Name^a^** | **Subcellular Location^b^** | **Protein Molecules per Cell^c^** | **Function^a,b^** |
| --- | --- | --- | --- | --- |
| Pdr5p | Yor153wp | PM | 42,000 | PDR network transporter; export of numerous chemicals (e.g. anticancer drugs, antibiotics, mycotoxins, azole antifungals). |
| Pdr10p | Yor328wp | PM | No data | PDR network transporter. |
| Pdr11p | Yil013cp | PM | No data | PDR network transporter; sterol uptake. |
| Pdr12p | Ypl058cp | PM | 752 | Export of weak organic acids (e.g. sorbate, benzoate). |
| Pdr15p | Ydr406wp | PM | No data | PDR network transporter; general stress response factor involved in cellular detoxification. |
| Pdr18p | Ynr070wp | PM | No data | PDR network transporter. |
| Adp1p | Ycr011cp | ERM | 339 | Unknown. |
| Aus1p | Yor011wp | PM | No data | Sterol uptake. |
| Snq2p | Ydr011wp | PM | 1,300 | PDR network transporter; export of singlet oxygen species (e.g. cercosporin). |
| Yor1p | Ygr281wp | PM | 3,610 | PDR network transporter; export of numerous organic anions (e.g. oligomycin). |
| Unidentified | Yol075cp | PM | No data | Unknown. |
| PM, Plasma Membrane; ERM, Endoplasmic Reticulum Membrane.  **References: ^a^** <http://www.yeastgenome.org/>, **^b^** http://www.uniprot.org/uniprot/, **^c^**Ghaemmaghami et al., 2003. | | | | |
