# Supplementary material for: Protein engineering of Saccharomyces cerevisiae transporter Pdr5p identifies key residues that impact Fusarium mycotoxin export and resistance to inhibition
Source: Microbiologyopen. 2016 Jun 4;5(6):979–91. doi: 10.1002/mbo3.381 (PMC5221463; doi:10.1002/mbo3.381)
Supplement: Supplementary file 6 — Table S2. Relative growth ratios of S1360 Pdr5p in FK506 or enniatin B. [file MBO3-5-979-s006.docx]

|  | FK506 | | Enniatin B | |
| --- | --- | --- | --- | --- |
| Yeast Strain | Relative Growth Ratio (%) ^a^ | SEM ^b^ | Relative Growth Ratio (%) | SEM |
| WT | 96.98 | 0.38 | 105.43 | 0.50 |
| S1360A | 95.00 | 0.20 | 104.33 | 0.24 |
| S1360C | 97.46 | 0.06 | 104.23 | 0.55 |
| S1360D | 100.32 | 1.09 | 110.00 | 1.13 |
| S1360E | 99.23 | 0.90 | 106.65 | 1.55 |
| S1360F | 95.80 | 0.77 | 101.73 | 2.78 |
| S1360G | 94.40 | 1.43 | 102.98 | 1.89 |
| S1360H | 94.86 | 1.11 | 103.10 | 2.12 |
| S1360I | 97.95 | 2.21 | 104.14 | 2.16 |
| S1360K | 95.40 | 0.68 | 106.79 | 0.72 |
| S1360L | 96.37 | 0.23 | 104.42 | 0.21 |
| S1360M | 95.98 | 1.11 | 103.43 | 1.15 |
| S1360N | 98.93 | 0.67 | 105.28 | 0.86 |
| S1360P | 98.12 | 1.07 | 107.00 | 0.87 |
| S1360Q | 97.24 | 0.19 | 103.04 | 0.33 |
| S1360R | 99.86 | 0.62 | 108.56 | 0.54 |
| S1360T | 99.85 | 1.66 | 102.33 | 2.25 |
| S1360V | 96.46 | 1.87 | 102.92 | 1.76 |
| S1360W | 97.50 | 0.18 | 106.68 | 0.86 |
| S1360Y | 95.53 | 0.12 | 103.29 | 0.28 |
| E | 93.96 | 0.49 | 103.55 | 0.56 |
| ^a^ Determined as described in Materials and Methods. All data are expressed as means (n≥3).  ^b^ ± Standard error of the mean. | | | | |
