# Supplementary material for: Protein engineering of Saccharomyces cerevisiae transporter Pdr5p identifies key residues that impact Fusarium mycotoxin export and resistance to inhibition
Source: Microbiologyopen. 2016 Jun 4;5(6):979–91. doi: 10.1002/mbo3.381 (PMC5221463; doi:10.1002/mbo3.381)
Supplement: Supplementary file 7 — Table S3. Relative growth ratios of T1364 Pdr5p in FK506 or enniatin B. [file MBO3-5-979-s007.docx]

|  | FK506 | | Enniatin B | |
| --- | --- | --- | --- | --- |
| Yeast Strain | Relative Growth Ratio (%) ^a^ | SEM ^b^ | Relative Growth Ratio (%) | SEM |
| WT | 97.56 | 0.66 | 105.66 | 0.70 |
| T1364A | 96.92 | 1.05 | 104.23 | 1.17 |
| T1364C | 98.81 | 0.78 | 105.73 | 0.76 |
| T1364D | 97.34 | 1.69 | 112.06 | 0.68 |
| T1364E | 98.21 | 0.86 | 107.61 | 1.50 |
| T1364F | 95.23 | 0.72 | 102.81 | 1.53 |
| T1364G | 97.27 | 0.82 | 104.02 | 0.81 |
| T1364H | 98.26 | 1.48 | 104.48 | 2.08 |
| T1364I | 95.81 | 0.38 | 104.03 | 1.07 |
| T1364K | 96.65 | 0.57 | 106.80 | 1.27 |
| T1364L | 93.80 | 0.62 | 103.54 | 0.52 |
| T1364M | 94.55 | 0.62 | 102.49 | 0.65 |
| T1364N | 98.66 | 0.55 | 105.50 | 0.07 |
| T1364P | 98.08 | 0.52 | 106.25 | 1.31 |
| T1364Q | 101.76 | 0.94 | 104.23 | 0.81 |
| T1364R | 90.78 | 1.52 | 106.46 | 0.72 |
| T1364S | 96.09 | 1.20 | 102.32 | 0.69 |
| T1364V | 94.06 | 0.88 | 100.19 | 0.44 |
| T1364W | 94.12 | 0.57 | 100.15 | 1.06 |
| T1364Y | 92.41 | 0.79 | 100.78 | 0.96 |
| E | 93.90 | 0.41 | 103.56 | 0.62 |
| ^a^ Determined as described in Materials and Methods. All data are expressed as means (n≥3).  ^b^ ± Standard error of the mean. | | | | |
